# Ghrelin, adipokines, metabolic factors in relation with weight status in school-children and results of a 1-year lifestyle intervention program

**DOI:** 10.1186/s12986-015-0039-9

**Published:** 2015-11-14

**Authors:** Christine Rambhojan, Elodie Bouaziz-Amar, Laurent Larifla, Jacqueline Deloumeaux, Josiane Clepier, Jean Plumasseau, Jean-Marc Lacorte, Lydia Foucan

**Affiliations:** Equipe de recherche sur le Risque Cardio métabolique, ECM/LAMIA EA4540, Université des Antilles et de la Guyane, Guadeloupe, France; Sorbonne Université, UPMC Univ Paris 06, UMR_S 1166, ICAN, F-75005 Paris, France; INSERM, UMR_S 1166, ICAN, F-75005 Paris, France; AP-HP, Hôpital Pitié-Salpêtrière-Charles Foix, Biochimie Endocrinienne et Oncologique, F-75651 Paris, Cedex France; Service de cardiologie, CHU de Guadeloupe, 97159 Pointe-à-Pitre, Guadeloupe, France; Réseau GRANDIR, Guadeloupe, France; Centre d’Examens de Santé, AGREXAM, Guadeloupe, France; Département de Santé Publique, CHU, 97159 Pointe-à-Pitre, Guadeloupe, FWI France

**Keywords:** Adolescents, Obesity, Adipokines, Ghrelin, Lifestyle intervention

## Abstract

**Background:**

Overweight in Guadeloupe is a public health matter affecting children and adults. In the present study we evaluated the metabolic profile, including serum ghrelin, leptin and adiponectin levels, in normal weight, overweight and obese school children and we analyzed the potential changes in anthropometric and metabolic risk factors after a 1-year lifestyle intervention program.

**Methods:**

Parameters were assessed at baseline and at 1 year. Three groups (G) were defined according the International Obesity Task Force reference values, G1: normal weight / G2: overweight / G3: obese. The lifestyle intervention included dietary counseling, regular physical activity and family support.

**Results:**

A total of 120 children (G1: *n* = 44, G2: *n* = 39, G3: *n* = 37), aged 11– 15 years and 59 % girls were enrolled. Obese children showed significant lower HDL-C, adiponectin and ghrelin concentrations, higher triglycerides, fasting blood glucose, insulin and leptin levels and also higher frequencies of abdominal obesity (G1: 2.3 %, G2: 28.2 %, G3: 73 %) and insulin resistance (GI: 39 %, G2: 72 %, G3: 89 %) than the other groups. In the overall sample, the linear regressions exploring the associations of ghrelin, adiponectin and leptin with age, gender, BMI z-score, HOMA-IR and tanner stage as independent variables showed strong associations of leptin levels with weight status and insulin resistance at baseline. The models accounted for 58 % of variability in leptin levels compared with 26 and 15 % for adiponectin and ghrelin levels respectively.

In 83 children who completed the program, significant decreases in BMI z-score in overweight and obese children were noted. Leptin levels decreased significantly only in the obese group whereas adiponectin concentrations increased significantly in the three groups,

In obese children, a significant correlation was found between changes in BMI Z-score, and changes in leptin levels (*r* = 0.39; *P* = 0.049) but not with changes in adiponectin levels.

**Conclusions:**

Abdominal obesity and insulin resistance were highly prevalent in obese children highlighting their risk of metabolic complications in adulthood. A 1-year long lifestyle intervention was associated with improvement in BMI z-score and metabolic parameters.

## Background

Obesity is a growing health problem worldwide. In the French Caribbean island of Guadeloupe, overweight and obesity were recently estimated at 23 and 9 % respectively among children aged 5–14 years [1]. In this island, a high prevalence of diabetes (8 %) is also observed in the adult population. The causes of obesity are multifactorial but the main factor contributing to excessive weight gain in children should be the imbalance between energy intake and energy expenditure. The presence of obesity in childhood is associated with adverse effects on health including metabolic complications in which numerous cytokines and hormones are involved.

**Fig. 1:**
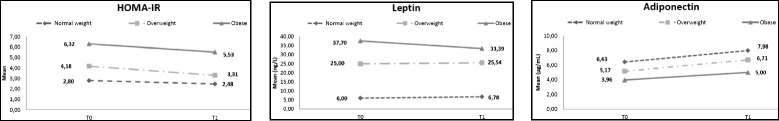
Changes in HOMA-IR index, leptin and adiponectin levels in normal-weight, overweight and obese children after a 1 year of lifestyle intervention. HOMA-IR normal weight: *P* = 0.065, overweight: *P* = 0.079, obese: *P* = 0.011. Leptin levels normal weight: *P* = 0.234, overweight: *P* = 0.857, obese: *P* = 0.008. Adiponectin levels normal weight: *P* = 0.001, overweight: *P* = 0.002, obese: *P* < 0.001

Among these cytokines, leptin and adiponectin, produced by adipose tissue, appear to play a role in glucose and lipid metabolism and energy homeostasis [2]. Ghrelin an orexigene hormone that also plays a role in energy metabolism by stimulating food intake and favoring weight and fat gain, has been recognized as an important regulator of glycemia and insulinemia [3].

Childhood obesity is a predictor of adult obesity and metabolic syndrome that are risk factors of morbidity and mortality [4–7] and, studies of long-term health consequences in adolescent males found that even moderate overweight is associated with excess mortality in adulthood [8].

Because lifestyle habits settle during childhood, it was recommended to precociously stop or prevent weight gain in overweight children notably by promoting lifestyle changes. These changes might also help to reduce obesity associated comorbidities.

Studies have analyzed the relationship between these hormones or cytokines and metabolic risk factors according to children's weight status [7, 9, 10]. But few studies have reported the concomitant variations of these factors after a lifestyle intervention [7, 11–13]. In addition, no data are available for these cytokines and metabolic risk markers in our children population.

Therefore, in the present study: 1) we evaluated the metabolic profile, including serum ghrelin, leptin and adiponectin levels, in schoolchildren classified as normal weight, overweight and obese and 2) we analyzed the potential changes in anthropometric and metabolic factors after a 1-year collaborative lifestyle intervention program.

## Methods

### Study design

We performed a cross-sectional analysis of basal data and a prospective study to evaluate after 1 year, the changes in children profiles in Guadeloupe Island (FWI).

### Study population

In 2013, children from a middle school were invited to participate in the study. They were eligible if they were i) aged between 11 and 15 years, ii) affiliated to a social security scheme and iii) in good health conditions. Exclusion criteria included seizure disorders, diabetes, cardiovascular disease or pregnancy.

The study was approved by the Ethic Committee (South West - Overseas III, Bordeaux, France) in October 2012 and the French National Agency for Medecine and Health Products Safety Security (ANSM). Written informed consent, to participate in this study, was obtained from all children and parents.

### Study protocol

At baseline, anthropometric and biochemical measurements were carried out in all children and, children and accompanying parents fulfilled questionnaires on lifestyle.

We conducted a 1-year lifestyle intervention program including 1) a nutritional information session presented, at the start of the study, by a nutritionist explaining healthy eating and nutrient needs to children and parents 2) Information on health risks for diseases related to lack of exercise and overweight 3) Encouragement for reduction of sedentary behaviors 4) Advices to achieve five hours per week of school physical education and/or physical activity outside of school and 5) Participation of parents.

The school usually provided three hours per week of physical education, supervised by physical education teachers.

Phone calls were made to maintain adherence to the program and, in voluntary children, anthropometric clinical and biological parameters (except ghrelin concentrations) were evaluated after the 1-year lifestyle intervention.

### Data collection

Clinical and biological examinations were performed at the referring Health Centre of Guadeloupe (AGREXAM). Participants were interviewed by a research Engineer and physicians using standard questionnaires that provided information on age, sex, anthropometric measurements, lifestyle and use of treatments.

Anthropometric measurements were performed by trained nurses. Height in centimeters (cm) and weight in kilograms (kg) were measured with participants standing without shoes and lightly clothed. The body mass index (BMI) was calculated as weight/height^2^ (kg/m^2^). The standard deviation score of body mass index (BMI Z-score) was used to provide a standard indicator of relative adiposity [14] and was calculated as [(measured value – average value in the reference population)/standard deviation in the reference population] using a “Pediatric z-Score Calculator”. Waist circumference (WC) in centimeters (cm) was measured in a standing position at the navel level at minimal respiration and the waist-to-height ratio (WHtR) was calculated.

Systolic and diastolic blood pressure (SBP and DBP, respectively) were assessed using automated monitors after resting for at least 5 min. The retained values were the average of two readings (left and right arms).

Pubertal stage was assessed according to Tanner [15]. Tanner stages 3, 4 and 5 defined pubertal/post-pubertal development.

### Laboratory measurements

#### Blood sample analysis

Blood samples were collected by venipuncture after an overnight fast. The biochemical analyses were performed in a single laboratory using the same methods.

Blood glucose level (mmol/L) was assessed using the glucose oxidase method. Serum lipid levels (mmol/L) were measured enzymatically.

Serum insulin levels (μIU/mL) were measured using the COBAS electro chemiluminescence immune assay test (Roche Diagnostics, Basel, Switzerland). The homeostasis model assessment to determined insulin resistance (HOMA-IR) was calculated using the following formula: [fasting insulin (μIU/mL) x fasting glucose (mmol/L)/22.5] [16].

Plasma samples were frozen at −80 °C until measurements of ghrelin, leptin and adiponectin and assayed in duplicate. Total ghrelin (ng/L) was measured with RIA kit Merck-Millipore® (EZGRT-89 K), leptin ng/mL and total adiponectin (μg/mL) were measured with ELISA kits Biovendor® (RD191001100) and Alpco® (47-ADPHUE-01) respectively. These measurements were made concomitantly for the samples collected at baseline and at one year (end of the study).

### Definition of factors

*Weight status* The children were classified according to the definition of overweight and obesity by the International Obesity Task Force (IOTF), that provide age and sex specific cut off points in childhood (from 2–18 years), predicting the accepted adult cut off points of a BMI of 25 and 30 kg/m^2^ (for overweight and obesity respectively) [17]. The three categories were defined as follows: normal weight with BMI < IOTF-25 kg/m^2^, overweight with BMI ≥ IOTF-25 kg/m^2^ and BMI < IOTF-30 kg/m^2^ and obesity.with BMI ≥ IOTF-30 kg/m^2^.

*Abdominal obesity* in children was defined as WHtR greater or equal to 0.5 [18].

*Blood lipid abnormalities* was defined as HDL-cholesterol <1.03 or TG ≥ 1.7 [18].

*Insulin resistance* was defined by a HOMA-IR > 3.16 [19].

### Statistical analysis

Data are presented as numbers (percentages) for categorical variables and as means ± standard deviations (SD) for continuous variables. The chi-squared test and ANCOVA with adjustment for age, sex were used to test percentage and mean differences between the three groups. Non parametric tests were used for comparison between data at baseline and 1-year.

The Pearson correlation test was used to study the relationships between serum ghrelin, leptin, adiponectin concentrations and the other continuous variables.

We also used the multivariate linear regressions in the overall study population to evaluate the associations of serum ghrelin, leptin and adiponectin as dependent variables and age, gender, weight status, Tanner stage and HOMA-IR as independent variables.

The IBM SPSS Statistics software version 21 was used for data analyses. All tests were two-sided and a *P* value < 0.05 was considered significant.

## Results

### Analysis of parameters at baseline

Overall 120 children of both sexes were enrolled in the study.

The accompanying parents were 80 % women and 20 % men. Forty four (37 %) of them were overweight/obese (BMI > 25 kg/m^2^) and 29 % were obese (BMI ≥ 30 kg/m^2^). Family history of diabetes was noted in 50 % of accompanying parents and 7 % had personal history of diabetes.

The children were categorized into three groups: group 1 (G1: normal weight; *n* = 44), group 2 (G2: overweight; *n* = 39) and group 3 (G3: obeses; *n* = 37). The characteristics of the study population according to weight status at baseline are presented in Table 1.

**Table 1:** Characteristics of school-children 11 to 15 years according to weight status at baseline

Parameters	Overall	Normal-weight	Overweight	Obese	*P*
	*n* =120	*n* =44	*n* =39	*n* =37	
Sex (girls)	59.2 %	52.3 %	69.3 %	56.8 %	*0.270*
Age (Years)	12.4 ± 1.1	12.2 ± 1.0	12.5 ± 1.2	12.7 ± 1.1	*0.130*
BMI (Kg/m^2^)	24.1 ± 6.1	18.4 ± 2.3***	24.2 ± 1.6^¥¥¥^	30.1 ± 4.9^§§§^	***<0.001***
BMI z-score	1.08 ± 1.18	−0.17 ± 1.04***	1.47 ± 0.23^¥¥¥^	2.15 ± 0.26^§§§^	***<0.001***
WC (cm)	73.9 ± 12.9	62.9 ± 7.1***	75.1 ± 6.5^¥¥¥^	86.6 ± 10.3^§§§^	***<0.001***
WHtR	0.46 ± 0.08	0.39 ± 0.04***	0.47 ± 0.04^¥¥¥^	0.53 ± 0.06^§§§^	***<0.001***
Abdominal obesity (%)	32.5 %	2.3 %***	28.2 %^¥¥^	73.0 %^§§§^	***<0.001***
Tanner stage ≥ 3 (pubertal/post pubertal)	92.5 %	90.9 %	92.3 %	94.6 %	*0.82*
SBP (mmHg)	113 ± 10	110 ± 9**	113 ± 8	116 ± 12	***0.015***
DBP (mmHg)	68 ± 8	66 ± 6	69 ± 9	70 ± 7	*0.126*
HDL-Cholesterol (mmol/L)	1.32 ± 0.35	1.52 ± 0.36***	1.22 ± 0.28^¥¥¥^	1.18 ± 0.28^§§§^	***<0.001***
HDL-Cholesterol < 1.03 mmol/L(%)	21.7 %	11.4 %	28.2 %^¥^	27.0 %	*0.261*
LDL-Cholesterol (mmol/L)	2.57 ± 0.67	2.55 ± 0.72	2.59 ± 0.62	2.57 ± 0.69	*0.970*
Triglycerides(mmol/L)	0.78 ± 0.44	0.66 ± 0.34***	0.74 ± 0.35	0.95 ± 0.57	***0.007***
Triglycerides ≥ 1.7 mmol/L (%)	4.2 %	0 %^*^	5.1 %	8.1 %	*0.287*
Fasting blood glucose (mmol/L)	4.8 ± 0.4	4.7 ± 0.4	4.8 ± 0.4	4.9 ± 0.4	***0.019***
Insulin (μIU/mL)	21.3 ± 12.9	14.6 ± 4.6***	21.7 ± 13.9^¥^	28.8 ± 14.3^§^	***<0.001***
HOMA-IR	4.6 ± 3.0	3.5 ± 1.1***	4.7 ± 3.0^¥^	6.5 ± 3.5^§^	***<0.001***
HOMA-IR > 3.16 (%)	65.0 %	38.6 %***	71.8 %^¥¥^	89.2 %	***<0.001***
Ghrelin (ng/L)	1379 ± 488	1541 ± 514^*^	1355 ± 440	1221 ± 457	***0.012***
Leptin ng/mL	22.9 ± 20.4	8.5 ± 6.1***	23.6 ± 12.6^¥¥¥^	39.6 ± 22.9^§§§^	***<0.001***
Adiponectin μg/mL	5.4 ± 2.6	6.8 ± 2.8***	4.9 ± 2.2^¥¥^	4.1 ± 1.8	***<0.001***

Data in this table are mean ± SD and column percentage

*BMI* body mass index, *WC* waist circumference, *WHtR* wait-to-height ratio, *SBP* systolic blood pressure, *DBP* diastolic blood pressure, *HDL-C* high density lipoprotein cholesterol, *LDL-C* low density lipoprotein cholesterol, *TG* triglycerides, *HOMA-IR* homeostasis model assessment-insulin resistance

*P*: *P* value for global comparison between the three groups

Comparison for normal-weight *vs* obese children * *P* < *0.05*, ** *P* < *0.01*, *** *P* < *0.001*

Comparison for normal-weight *vs* overweight children ^¥^ *P* < *0.05*, ^¥¥^ *P* < *0.01*, ^¥¥¥^ *P* < *0.001*

Comparison for overweight *vs* obese children: ^§^ *P* < *0.05*, ^§§^ *P* < *0.01*, ^§§§^ *P* < *0.001*

All p-values are in italic and significant p-value are in bolditalic

The mean age of the study population was 12.4 ± 1.1 years and 59 % were girls. Tanner stages were distributed as follows: stage 2: 7.5 %, stage 3: 58.3 %, stage 4: 30 % and stage 5: 4.2 %.

Overall, 92.5 % of the children had a tanner stage ≥ 3 (pubertal/post pubertal development) with no significant difference between the three groups (*P* = 0.82, Table 1). The frequency of children eating at school canteen was higher in normal weight (53 %) than in overweight (28 %) and obese (30 %) children; (*P* = 0.027), (data not shown).

In accordance with the design of the study, all the anthropometric parameters (BMI, BMI z-score, WC and WC/height) were significantly different between the three groups (*P* < 0.001 for all parameters). Obese children had higher SBP (*P* = 0.015), triglycerides (*P* = 0.007), fasting blood glucose (*P* = 0.019), insulin (*P* < 0.001) and leptin levels (*P* < 0.001), whereas they had lower HDL-C (*P* < 0.001), ghrelin (*P* = 0.001) and adiponectin levels (*P* < 0.001) than the other groups. Abdominal obesity and insulin resistance were also more frequently found in the obese group (*P* < 0.001 for both).

Leptin levels were higher in girls than in boys in the overall study sample (27 *vs* 16 ng/mL; *P* = 0.004) whereas no significant gender differences were found for adiponectin (*P* = 0.164) and ghrelin levels (*P* =0.110).

The correlations between leptin, ghrelin, adiponectin levels and the other continuous variables: anthropometric parameters, blood lipids and insulin levels, among boys and among girls respectively are presented in Table 2a and b. Leptin, ghrelin, adiponectin levels were significantly correlated with the three anthropometric parameters (BMI z score, WC, WHt) in both genders except for ghrelin in girls. Concerning FBG and blood lipid parameters, in boys, significant correlations were found except for ghrelin with TG levels, for leptin with FBG and LDL-C and for adiponectin with FBG, LDL-C and TG levels. In girls, no significant correlation was found for ghrelin with the 4 parameters, for leptin with LDL-C and for adiponectin with LDL-C and TG.

**Table 2:** Correlations between Ghrelin, Adiponectin and Leptin and anthropometric and metabolic parameters among school-children by gender 11 to 15 years at baseline

Parameters	2a: Boys						2b: Girls					
	Ghrelin (ng/L)		Leptin(ng/mL)		Adiponectin (μg/mL)		Ghrelin (ng/L)		Leptin(ng/mL)		Adiponectin (μg/mL)	
	r	*P*	r	*P*	r	*P*	r	*P*	r	*P*	r	*P*
BMI z-score	−0.59	***<0.001***	0.70	***<0.001***	−0.40	***0.005***	−0.08	*0.523*	0.66	***<0.001***	−0.40	***0.001***
WC (cm)	−0.64	***<0.001***	0.78	***<0.001***	−0.46	***0.001***	−0.12	*0.313*	0.79	***<0.001***	−0.46	***<0.001***
WHtR	−0.51	***<0.001***	0.85	***<0.001***	−0.36	***0.013***	−0.10	*0.399*	0.73	***<0.001***	−0.50	***<0.001***
FBG (mmol/L)	−0.37	***0.001***	0.25	*0.087*	−0.10	*0.483*	0.07	*0.587*	0.36	***0.003***	−0.32	***0.006***
HDL-C (mmol/L)	0.41	***0.005***	−0.36	***0.013***	0.57	***<0.001***	0.16	*0.190*	−0.39	***0.001***	0.46	***<0.001***
LDL-C (mmol/L)	0.36	***0.014***	0.28	*0.058*	0.11	*0.485*	0.17	*0.149*	−0.05	*0.707*	−0.02	*0.881*
TG (mmol/L)	−0.10	*0.492*	0.53	***<0.001***	−0.18	*0.238*	0.21	*0.076*	0.25	***0.038***	−0.07	*0.585*
Insulin (μU/mL)	−0.52	***<0.001***	0.55	***<0.001***	−0.44	***0.002***	−0.13	*0.267*	0.47	***<0.001***	−0.35	***0.003***
HOMA-IR index	−0.53	***<0.001***	0.57	***<0.001***	−0.44	***0.002***	−0.11	*0.357*	0.50	***<0.001***	−0.36	***0.003***

*BMI* body mass index, *WC* waist circumference, *WHtR* wait-to-height ratio, *FBG* Fasting blood glucose, *HDL-C* high density lipoprotein cholesterol, *LDL-C* low density lipoprotein cholesterol, *TG* triglycerides, *HOMA-IR* homeostasis model assessment-insulin resistance

All p-values are in italic and significant p-value are in bolditalic

High significant correlations were also noted between ghrelin, leptin and adiponectin levels with insulin levels and HOMA-IR in both genders except for ghrelin levels in girls.

All anthropometric parameters were positively correlated with insulin and HOMA-IR in both genders (data not shown).

In considering the weight status of accompanying parents, insulin resistance (HOMA IR > 3.16) was noted in 61 % of children from parents with normal weight and in 70 % of children from overweight or obese parents (data not shown).

Table 3 presents the results of the multivariate linear regression analyses exploring the associations of ghrelin, adiponectin and leptin with age, gender, weight status, HOMA-IR and tanner stage as independent variables, at baseline.

**Table 3:** Multivariate linear regression for Ghrelin, Adiponectin and Leptin levels in 120 schoolchildren 11 to 15 years, at baseline

	Model 1 Ghrelin (ng/L)		Model 2 – Adiponectin (μg/mL)		Model 3 Leptin (ng/mL)	
	*r* ^2^ = 0.15		*r* ^2^ = 0.26		*r* ^2^ = 0.58	
	Adjusted *r* ^2^ = 0.11		Adjusted *r* ^2^ = 0.22		Adjusted *r* ^2^ = 0.56	
Variables	nSRC (SE)	*P*	nSRC (SE)	*P*	nSRC (SE)	*P*
Age (Years)	−113 (88)	*0.205*	0.44 (0.44)	*0.318*	3.18 (2.61)	*0.226*
Gender (girls)	−71 (102)	*0.488*	0.68 (0.51)	*0.187*	7.87 (3.02)	***0.010***
Weight Status						
Overweight	−81 (109)	*0.455*	−1.67 (0.53)	***0.002***	13.88 (3.15)	***<0.001***
Obese	−164 (119)	*0.171*	−2.10 (0.59)	***0.001***	26.36 (3.49)	***<0.001***
Tanner stage 2 to 5	52 (135)	*0.689*	−0.45 (0.68)	*0.508*	−0.73 (4.01)	*0.856*
HOMA-IR	−29 (16)	*0.071*	−0.21 (0.08)	***0.010***	1.50 (0.47)	***0.002***

*nSRC* non standardized regression coefficient, *SE* standard error, *HOMA-IR* homeostasis model assessment forinsulin resistance

All p-values are in italic and significant p-value are in bolditalic

In model 1, for ghrelin levels, no significant association was noted with the five variables but association with HOMA-IR was nearly significant (*P* = 0.070). The model accounted for 15 % (*r*^2^ = 0.15) of the variability in ghrelin levels.

In model 2, for adiponectin levels, significant negative associations were noted with overweight (*P* = 0.002), obesity (*P* = 0.001) and HOMA-IR (*P* = 0.010) and the model accounted for 26 % (*r*^2^ = 0.26) of the variability in adiponectin levels.

In model 3, for leptin levels, significant positive associations were noted with gender (*P* = 0.011), overweight (*P* < 0.001), obesity (*P* < 0.001) and HOMA-IR (*P* = 0.002). The average increase in leptin levels was 7.9 ng/dL for gender (girls), 13.9 ng/dL for overweight, 26.4 ng/dL for obesity and 1.50 ng/dL for each unit of HOMA-IR. This multivariate model accounted for 58 % (*r*^2^ = 0.58) of the variability in leptin levels.

No significant average change in ghrelin, leptin and adiponectin levels was noted in relation with tanner stage.

Table 4 presents changes in clinical and metabolic parameters at one year of follow-up. Of the 120 children who participated to the study at baseline, 83 have accepted to undergo evaluation for clinical and biological parameters after one year (28 in normal weight, 28 in overweight, and 27 in obese children).

**Table 4:** Clinical and biological parameters in 83 children at baseline and after 1 year of a lifestyle intervention program

Parameters	Normal-weight (*n* = 28)			Overweight (*n* = 28)			Obese (*n* = 27)		
	Baseline	T1-year	*P*	Baseline	T1-year	*P*	Baseline	T1-year	*P*
BMI z-score	−0.10 ± 1.03	−0.16 ± 0.85	*0.446*	1.47 ± 0.24	1.33 ± 0.44	***0.021***	2.18 ± 0.27	2.08 ± 0.40	***0.008***
FBG (mmol/L)	4.67 ± 0.38	4.63 ± 0.34	*0.690*	4.82 ± 0.42	4.69 ± 0.42	***0.047***	4.95 ± 0.40	4.69 ± 0.35	***0.002***
HDL-C (mmol/L)	1.59 ± 0.36	1.45 ± 0.31	***<0.001***	1.21 ± 0.28	1.18 ± 0.21	*0.425*	1.19 ± 0.29	1.14 ± 0.28	*0.248*
LDL-C (mmol/L)	2.61 ± 0.75	2.37 ± 0.66	***0.018***	2.68 ± 0.59	2.48 ± 0.48	***0.015***	2.46 ± 0.59	2.43 ± 0.47	*0.410*
TG (mmol/L)	0.55 ± 0.26	0.56 ± 0.24	*0.945*	0.78 ± 0.40	0.73 ± 0.39	*0.682*	0.86 ± 0.32	0.80 ± 0.36	*0.254*
Insulin (μU/mL)	13.4 ± 3.6	11.8 ± 6.1	***0.048***	19.9 ± 12.5	15.8 ± 7.0	***0.019***	28.4 ± 13.7	26.2 ± 17.7	***0.019***
HOMA-IR index	2.80 ± 0.86	2.48 ± 1.37	*0.065*	4.18 ± 3.04	3.31 ± 1.56	*0.079*	6.32 ± 3.19	5.53 ± 3.95	***0.011***
Leptin (ng/mL)	6.0 ± 5.19	6.78 ± 1.9	*0.234*	25.0 ± 13.0	25.5 ± 18.1	*0.857*	37.7 ± 22.5	33.4 ± 24.2	***0.008***
Adiponectin (μg/mL)	6.43 ± 2.51	7.98 ± 3.56	***0.001***	5.17 ± 2.39	6.71 ± 2.77	***0.002***	3.96 ± 1.84	5.0 ± 2.41	***<0.001***

*FBG* fasting blood glucose, *BMI* body mass index, *WC* waist circumference, *HOMA-IR* homeostasis model assessment for insulin resistance

All p-values are in italic and significant p-value are in bolditalic

Mean BMI z-score significantly decreased in overweight (1.47 to 1.33; *P* = 0.021) and obese children (2.18 to 2.08; *P* = 0.008) whereas, no significant change was noted in the normal weight group (−0.10 to −0.16; *P* = 0.446) (Table 4).

Concerning the metabolic parameters, significant decreases were observed in normal weight children for LDL-C (*P* = 0.018) and insulin levels (*P* =0.048). The HDL-C levels decreased in this group (*P* <0.001) but remained higher than in the other groups. Fasting blood glucose (*P* = 0.047), LDL-C (*P* = 0.015) and insulin levels (*P* = 0.019) improved in overweight children. In obese children, significant improvements were also observed in FBG (*P* = 0.002), insulin levels (*P* = 0.019) and HOMA-IR (*P* = 0.011). Significant rises in adiponectin levels were observed in normal weight overweight and obese children (*P* = 0.001, *P* = 0.002, *P* < 0.001 respectively). Leptin levels remained unchanged at 1 year of follow-up in normal weight and overweight children but decreased significantly in obese children (*P* = 0.008). Table 4 and fig. 1 present changes in clinical and metabolic parameters at one year of follow-up.

Combining the results of the three groups, a nearly significant correlation was found between changes in BMI z-score, and changes in leptin concentrations (*r* = 0.20; *P* = 0.060). This positive correlation was significant in the obese group (*r* = 0.39; *P* = 0.049). Conversely no significant correlation was found between changes in BMI z-score, and both changes in HOMA-IR and adiponectin levels (data not shown).

We evaluated the frequencies of children undergoing concomitant changes in the three metabolic parameters at 1 year (decrease HOMA IR and leptin levels and increase adiponectin levels) and we found a frequency of 27 % in children from parents with normal weight and of 37 % in children from overweight or obese parents.

## Discussion

The current study is the first to evaluate the metabolic profile in overweight and obese Guadeloupean children. Obesity was associated with an adverse metabolic profile including, high frequencies of abdominal obesity (73 %) and of insulin resistance (89 %) highlighting the risk for development of type 2 diabetes and future cardiovascular complications in adulthood [4, 20]. These obese children also exhibited, at baseline, the highest values for triglycerides and leptin levels and the lowest values for HDL-cholesterol, ghrelin and adiponectin levels. In addition, leptin levels appeared to have a stronger relationship with weight status and HOMA-IR than ghrelin and adiponectin at baseline which is in favor of a role of leptin in body weight regulation [21]. Our 1-year collaborative lifestyle intervention, led to improvements in BMI z-score and in most of the metabolic parameters particularly in the obese children. The results, after the intervention, also showed that changes in BMI z-score correlated with changes in leptin levels. Leptin levels decreased significantly only in the obese group whereas adiponectin concentrations increased in normal weight, overweight and obese children.

### Anthropometric parameters

All the anthropometric parameters (WC, WHtR and BMI Z-score) were significantly correlated with ghrelin, leptin and adiponectin except in girls in whom no correlation with ghrelin was observed. Abdominal obesity, defined in children as WHtR greater than 0.5 [18], has been associated in adulthood with cardio vascular complications [18] and was found in 2.3 % of normal weight, 28.2 % of overweight and 73 % of our obese children.

In a recent review, the authors recommended to pay more attention to abdominal obesity in children and adolescents because central body fat deposition increases the risk of cardio-metabolic risk factors, whatever the definition used for abdominal obesity and whatever the methods used for anthropometric measurements, [22].

### Metabolic profile, ghrelin and cytokines

The lowest HDL-C concentrations, the highest triglycerides and FBG concentrations and the highest values for HOMA-IR index were observed in the obese children. It is well known that obesity is associated with comorbidities such as dyslipidemia, hypertension, insulin resistance [20, 23] and with early indication of atherosclerosis [24, 25]. We also observed high frequencies of insulin resistance (defined as HOMA-IR greater than 3.16) in obese (89 %), overweight (72 %) and normal weight children (39 %). Insulin sensitivity index was previously reported lower in black compared with white adolescents suggesting a more diabetic profile in the first group [26]. In our general population in which most people are of African ancestry and diabetes is highly prevalent (8 %), the results observed in children and adolescents highlight their high risk of metabolic complications. This risk is probably increased due to children family history showing 50 % of overweight and 7 % of diabetes in accompanying parents.

Despite ghrelin levels were not significantly associated with HOMA-IR in the linear regression (Table 3), the hormone has been recognized as an important regulator of energy metabolism and serves as a physiological regulator of insulinemia and glycemia [3].

Adipose tissue is considered as an endocrine organ producing several proteins (including leptin and adiponectin) with important biological activity and, the adipocyte plays a central role in the balance, or imbalance, of metabolic homeostasis [27]. Adiponectin levels were the lowest and leptin levels the highest in the obese group. Numerous studies described significant negative correlations between adiponectin and the parameters of obesity. Low adiponectin concentrations which has been previously associated with coronary artery disease in the adult population of Guadeloupe [28] correlated with high plasma insulin levels and adverse blood lipid profile in the present study in children. Lower adiponectin levels in obesity were also associated with chronic inflammation, endothelial dysfunction, and insulin resistance [29, 30]. The three multivariate linear regression models for ghrelin, adiponectin and leptin levels, as dependent variables (Table 3) and age, gender, weight status, tanner stage and HOMA-IR as independent variables, highlighted the strong associations of leptin and adiponectin levels with body weight and insulin resistance at baseline. The model accounted for 58 % of variability in leptin levels compared with 26 and 15 % of variability in adiponectin and in ghrelin levels respectively. Leptin levels increased significantly by 1.50 ng/ml for each unit of HOMA-IR. These strong relationships were also reported in a study on 321 children (109 normal, 212 obese) aged 6–12 years, analysing leptin, ghrelin, adiponectin levels [10]. In this study, leptin was found as the most sensitive adipokine marker for predicting the accumulation of cardiovascular risk factors and the presence of metabolic syndrome [10]. The gender difference for leptin levels, previously reported in children and adults [7, 21, 31–33], was also found in our study with girls exhibiting higher values than boys. This difference might reflect the higher ratio of subcutaneous to visceral fat in women than in men since serum leptin is strongly related to subcutaneous fat mass [21]. But, other factors besides adiposity, such as sex steroids, might play a role in this gender difference [21].

Leptin administration has been demonstrated to reduce obesity in leptin-deficient *ob/ob* mice [34] and, to decrease food intake, body weight, increase energy expenditure and improve metabolic status in leptin deficient subjects [35, 36]. But, in most individuals, serum leptin levels are positively correlated with BMI and with indexes of obesity. Thus, the concept of leptin resistance has emerged to explain the paradoxical elevated leptin levels in obesity. Several mechanisms have been suggested to explain this leptin resistance, notably the impaired transport of leptin across blood brain barrier [37], or decrease of hypothalamic leptin receptor expression [38] as well as attenuation of leptin signaling by protein tyrosine phosphatases [39].

The mechanism by which increased body fat cause increased metabolic comorbidities is not really known. It was suggested that excess adipose tissue contribute to a state of chronic inflammation which promotes development of insulin resistance as well as other metabolic complications [40]. Leptin and adiponectin show opposite effects on inflammation and insulin resistance. While high leptin levels increase the expression of pro-inflammatory and pro-angiogenic factors [41] adiponectin, improves peripheral insulin sensitivity, and induces the production of anti-inflammatory cytokines [42].

### Changes in anthropometric and metabolic parameters after 1 year follow-up

In this study population without significant differences between groups in age, sex and Tanner stage distributions, we observed after the 1-year lifestyle intervention, a reducing in BMI z-score in overweight and obese children and improvements in most of the metabolic parameters with greater benefits in the obese group. This group showed significant improvements in fasting insulin levels, HOMA-IR, leptin and adiponectin levels. These results are interesting since increase of insulin levels, HOMA-IR and leptin levels would be expected after one year with increasing age or puberty. In fact, in EarlyBird, a prospective cohort study of healthy children, insulin resistance started to rise in mid-childhood, and HOMA-IR rose, almost linearly, from age of 8 [43]. Ballabriga A et al. reported that both prepubertal boys and girls showed a progressive increase of leptin levels during the years prior to the onset of puberty and until Tanner's stage 11 [44].

Serum leptin is believed to play a role in obesity but, although leptin levels were strongly associated with HOMA-IR and weight status at baseline, in the present study, significant decrease in leptin levels were noted only in obese children at one year follow-up. In other studies, after lifestyle intervention or after exercise training program, leptin levels decreased [45] or remained unchanged [7, 13, 46]. In addition, leptin levels increased in children without weight loss in one of these studies [13].

At one year of follow-up, adiponectin levels increased significantly in the three groups although normal weight children showed no significant reduction of BMI z-score. Cambuli et al. evaluated adipokines levels but also fat-free mass and fat mass, before and after 1 year of lifestyle intervention in 104 overweight and obese children [13]. In this study, adiponectin in particular increased from baseline at the end of the study. In addition, the authors found that changes in adiponectin were significantly and independently associated with changes in percent fat mass and that, even those children who did not lose weight obtained a significant reduction in percent fat mass [13]. It was previously reported that high adiponectin concentrations predicted lower prevalence of type 2 diabetes in obese Mexican children [47] and that low adiponectin concentrations were associated with increased risk of type 2 diabetes in adults [48, 49]. But, high leptin levels were not associated with risk of incident type 2 diabetes in an Aboriginal Canadian population [48]. In our population having a high prevalence of type 2 diabetes, adiponectin could be a tool for monitoring overweight or obese children having marked insulin-resistance.

### Implications in prevention of overweight in children

The efficiency of lifestyle intervention for childhood obesity has been previously reported with changes in body weight but also changes in metabolic parameters. Moreover, obesity treatment programs are more effective in reducing overweight in children if parents are involved. The positive effect of eating in school canteen should be highlighted. Our children with normal weight ate more frequently in school canteen than the other groups. In fact, it is a beneficial factor since the menus follow the recommendations designed to lower fat and sugar and increase vegetables and fruit in meals. In addition, the children can take advantage of the infrastructure for physical activity and sport activities proposed by the school.

### Limitation and strengths of the study

The potential limitations of our study include i) the small sample size even if other lifestyle intervention studies were conducted on small sample sizes [7, 12, 13] and ii) the absence of controls groups (without lifestyle intervention) that did not let us draw any causality link between the lifestyle intervention and the improvement of the parameters studied.

Nevertheless, the present study has a number of strengths. i) the children were healthy, comparable between groups for age, sex and Tanner stage distributions and presented no associated diseases that could influence the metabolic profile, ii) the use of traditional markers for metabolic disease (ie, lipid profile, insulin levels, HOMA-IR) and the measurement for the first time of adiponectin, leptin and ghrelin concentrations in a sample of Guadeloupean children and iii) the providing of data on changes in metabolic parameters at the end of the lifestyle intervention.

## Conclusion

Abdominal obesity and insulin resistance, that could precede other metabolic complications, were frequent in obese children highlighting their high risk of obesity and type 2 diabetes in adulthood. The 1-year collaborative lifestyle intervention, involving children parents and teachers seems rather beneficial, notably in obese children, with improvements in body composition, insulin, leptin and adiponectin levels. This improvement is probably the result of different factors including the fight against sedentary lifestyles and changes in eating habits in the families.

These results might be used to encourage parents to participate in prevention programs against overweight. In addition, sensitization of families (children and accompanying) on health risks related to sedentary behaviors must be continuous.

## Abbreviations

BMI: Body mass index
BMI z-score: Standard deviation score of body mass index
BP: Blood pressure
FBG: Fasting blood glucose
HDL-C: High-density lipoprotein Cholesterol
HOMA-IR: Homeostasis model assessment for insulin resistance
IOTF: International Obesity Task Force
LDL-C: Low-density lipoprotein Cholesterol
MetS: Metabolic syndrome
SD: Standard deviation
TG: Triglycerides
WC: Waist circumference
WHtR: Waist-to-height ratio
